# Molecular breeding in *Brassica* for salt tolerance: importance of microsatellite (SSR) markers for molecular breeding in *Brassica*

**DOI:** 10.3389/fpls.2015.00688

**Published:** 2015-09-04

**Authors:** Manu Kumar, Ju-Young Choi, Nisha Kumari, Ashwani Pareek, Seong-Ryong Kim

**Affiliations:** ^1^Plant Molecular Biology Laboratory, Department of Life Science, Sogang University, SeoulSouth Korea; ^2^College of Medicine, Seoul National University, SeoulSouth Korea; ^3^Stress Physiology and Molecular Biology Laboratory, School of Life Science, Jawaharlal Nehru University, New DelhiIndia

**Keywords:** *Brassica*, salt stress, abiotic stress, SSR markers, QTL

## Abstract

Salinity is one of the important abiotic factors for any crop management in irrigated as well as rainfed areas, which leads to poor harvests. This yield reduction in salt affected soils can be overcome by improving salt tolerance in crops or by soil reclamation. Salty soils can be reclaimed by leaching the salt or by cultivation of salt tolerance crops. Salt tolerance is a quantitative trait controlled by several genes. Poor knowledge about mechanism of its inheritance makes slow progress in its introgression into target crops. *Brassica* is known to be a good reclamation crop. Inter and intra specific variation within *Brassica* species shows potential of molecular breeding to raise salinity tolerant genotypes. Among the various molecular markers, SSR markers are getting high attention, since they are randomly sparsed, highly variable and show co-dominant inheritance. Furthermore, as sequencing techniques are improving and softwares to find SSR markers are being developed, SSR markers technology is also evolving rapidly. Comparative SSR marker studies targeting *Arabidopsis thaliana* and *Brassica* species which lie in the same family will further aid in studying the salt tolerance related QTLs and subsequent identification of the “candidate genes” and finding out the origin of important QTLs. Although, there are a few reports on molecular breeding for improving salt tolerance using molecular markers in *Brassica* species, usage of SSR markers has a big potential to improve salt tolerance in *Brassica* crops. In order to obtain best harvests, role of SSR marker driven breeding approaches play important role and it has been discussed in this review especially for the introgression of salt tolerance traits in crops.

## Introduction

Salinity in the soil is one of the serious obstacles for agriculture, due to which large areas of the agricultural lands are becoming unfertile. Three fourth of the total Earth surface is covered by saline water and hence significant proportion of this Earth is affected by saline conditions. Over 830 million hectares of land area in the entire earth are salt affected, either by saline water (403 million hectares) or by the conditions related with sodicity (434 million hectares; FAO, 2008) and it is more than six percent of the entire land area in the world. An excess amount of NaCl occurs as an abiotic environmental factor in many places such as salt deserts in the arid and semi-arid areas, coastal salt marshes and inland saline lakes (Kumar, 2013). During the last decades, apart from the natural salinity, salinization of soils due to intensive agriculture and irrigation has also been becoming a major problem in agriculture. When plants are exposed to salinity, it causes ion imbalance, ion toxicity and hyper osmotic stress (Yamaguchi and Blumwald, 2005; Kumar et al., 2013, 2014). It severely retards the crop growth and productivity. For most of the crops concentrations of 150 mM NaCl are highly toxic though, for a few crops, as low as 25 mM NaCl is lethal. Two main courses of actions were given special importance for providing the solution for salinity stress problem (Epstein, 1985; Ashraf, 1994; Flowers and Yeo, 1995; Grieve et al., 1999), which includes reclamation of saline soils by use of chemicals or by growing salt tolerant plants in the saline soils. Considering its low cost, feasible, and efficient approach, the latter strategy was being emphasized by many plant scientists during the past few decades. This includes cereals, legumes and other commercially important crops.

Apart from the cereals and legumes, oil seeds are very important for human food and are at the third position among the crops. At least forty different plant species are known to be grown for the oils production (Weiss, 1983). Among the oilseed crops, *Brassicas* which belongs to the family *Brassicaceae* are very important oilseed crops. The family *Brassicaceae* includes various crops, which are rich in nutritional and economic values. The members of the *Brassica* genus are sometimes collectively called as cabbages/mustards/-cole crops. *Brassica* contains more number of important horticultural and agricultural crops. The members of the *Brassica* genus also contains more number of weed species and wild relatives, making it a perfect platform for crop improvement practices, due to the presence of wide genetic base. Apart from the oilseeds (mustard seed, oilseed rape), almost every part of the plant of some species or the other are edible and grown for food, which includes the stems (kohlrali), root (Swedes, turnips), flower (Cualiflower, broccoli), and leaves (Cabbages, Brussels sprout). *Brassica* vegetables are most commonly regarded for their nutritional and medicinal properties (Beecher, 1994; Carvalhoa et al., 2006). They contain high amounts of soluble fiber and vitamin C (Divisi et al., 2006). *Brassica* contains different nutrients having potential anticancer properties like 3, 3′ diindolylmethane, sulforaphane, and selenium (Finley et al., 2005; Banerjee et al., 2012). Since *Brassicas* are of high agricultural importance, they are of much scientific interest.

Before discussing the markers and salinity tolerance in *Brassica*, it is important to know the relationship with in the *Brassica* species.

## Relationships between Crop *Brassicas*

**FIGURE 1:**
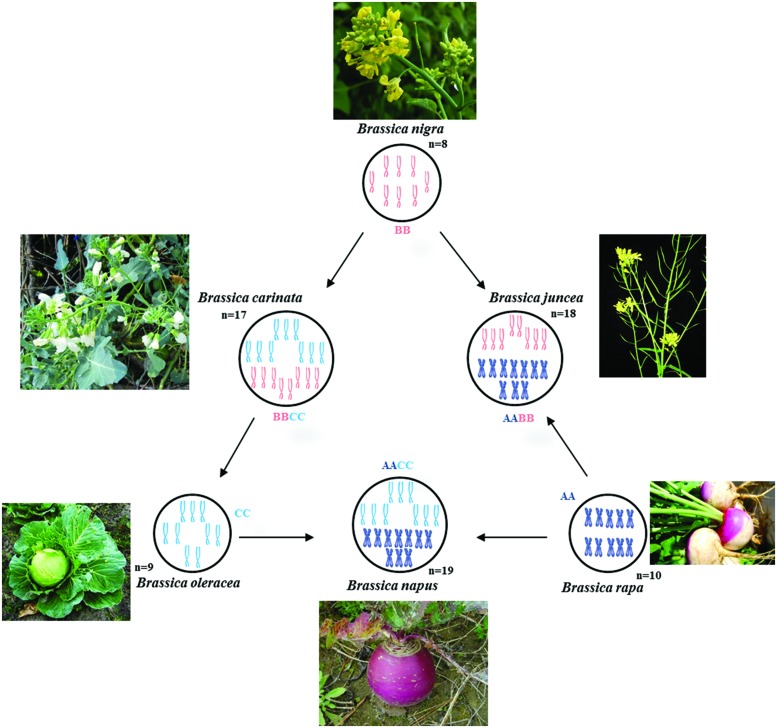
**Triangle of U diagram illustrating the genetic relationships between six species of the genus *Brassica*, such as Chinese cabbage (AA), Cabbage (CC), Rapeseed (AACC), Indian mustard (AABB), and other common vegetables, showing the genetic relationships between Chromosomes from each of the genomes A, B, and C are represented by different colors**.

The relationship between six particular species in *Brassica* genus (*B. carinata*, *B. juncea*, *B. napus*, *B. nigra*, *B. oleraceae*, and *B. rapa*) is well described by the Triangle of U theory (Nagaharu, 1935; **Figure 1**). The Triangle of U theory explains the high chromosome number species [*B. carinata* (BBCC), 2*n* = 34; *B. juncea* (AABB), 2*n* = 36; and *B. napus* (AACC), 2*n* = 38] which are amphidiploids and possibly formed through the interspecific hybridization between the low chromosome number species in pairs [*B. nigra* (BB), 2*n* = 16; *B. oleraceae* (CC), 2*n* = 18; and *B. rapa* (AA), 2*n* = 20].

## Salinity Tolerance in *Brassica*

Salt stress tolerance is one of the highly complex processes in number of plant species. Many complex mechanisms are involved at different plant developmental levels. But some of these mechanisms are functional at a particular time in a given species. Apart from this, the effect of one process can exclude the effect of the other process at a particular time (Gorham et al., 1991; Ashraf, 1994; Yeo, 1998; Carvajal et al., 1999). Salt stress tolerance in plants is a developmentally regulated phenomenon and tolerance at one stage of development sometimes may not correlate with the stress tolerance at other stages. For example, in barely, corn, rice, tomato, and wheat, salt stress tolerance tends to increase as the plants become older. The situation becomes more complicated with polyploidy species in comparison with their respective diploid parents. Polyploid species can withstand the harmful environmental factors such as salt stress tolerance better than their own respective diploid parents. Different studies gives the indications that the amphidiploid species, *Brassica carinata*, *B. juncea*, and *B. napus* have the superiority over the diploid species, *B. campestries*, *B. nigra*, and *B. oleraceae* in terms of salinity tolerance (Ashraf et al., 2001). It was also found that the amphidiploid genotypes, *B. carinata* and *B. napus* were salt stress tolerant when compared with *B. campestries*. Another amphidiploid *B. juncea* is intermediate in salt stress tolerance (Ashraf and McNeilly, 1990). The continued survival of salt stress tolerant plants and the differences between the genotypes with salt sensitive plant species point out the presence of a genetic basis of salt stress tolerance.

## Utilization of *Brassica* Genetic Diversity

In the presence of environmental stresses, such as drought, salt, cold, nutrient deficiency, and water logging, growth of the *Brassica* plants, their oil production and reproduction capabilities are reduced. Hence, these *Brassica* species are normally grown on standard non-saline conditions in order to maximize the yield. If they are grown on salt affected soils, yield losses are severe. Therefore, improvement of their salt stress tolerance is of considerable economic value. For breeding program to be successful, presence of a significant heritable distinction with in the gene pool of these crops is a compulsory requirement (Becker et al., 1995; O’Neill et al., 2003; Kumar, 2015). Because of the close relationship and the presences of important inter and intra specific distinctions within Brassica species, the breeding programs for salinity stress tolerance have been highly benefited. It is also believed that support from other approaches such as mutagenesis, fusion of protoplast or recombinant technology can also be helpful in achieving the desired target (Diers et al., 1996; Riaz et al., 2001; Seyis et al., 2003). Even though there is a great inter and intra specific variation for salt stress tolerance within *Brassica* species, generation of new variation through induced mutation and utilization of those new variants gives more scope for enhancing salt stress tolerance. Due to the advancements of molecular techniques, the mutants can be identified and analyzed using DNA fingerprinting and mapping on PCR based markers such as SSR, RAPD, AFLP, and STMS (Diers and Osborn, 1994; Halldén et al., 1994; Thormann et al., 1994; Plieske and Struss, 2001a).

## Molecular Markers in Breeding for Salinity Tolerance

Molecular genetics is one of the most important technologies in the today’s world. Stress tolerance and yield are difficult to breed using conventional methods because of their polygenic nature and are also largely influenced by environment and genotype. The complex quantitative feature of the most mechanisms involved in the salt stress tolerance is the main reason for the limited success of the modern salt tolerance breeding approaches (Yeo and Flowers, 1986). The association and application of the indirect selection markers which are genetically linked with the trait (s) of interest is a well-known approach for the betterment of the crop having difficult traits which includes salt stress tolerance (Im et al., 2014a). DNA marker technology has revolutionized the genome research and breeding in the recent decades. Implementation of various available markers and QTL mapping techniques have contributed for the good knowledge of genetic bases of various agriculturally significant traits such as resistance to biotic stresses, abiotic stresses tolerance, yield and nutritional quality in various crops (Xue et al., 2010; Ali et al., 2013).

Since, breeders use QTL linked markers to find the position of markers on the loci that controls the concerned traits; the number of methods to identify the phenotype is reduced. Therefore, the necessity for large-scale methods over time and space is significantly reduced. A few salt stress related QTL detected by SSR markers have been listed in the **Table 1**.

**Table 1:** Identification of quantitative trait loci (QTLs) by SSR markers for salt tolerance (ST) in different plant species.

Crop plants	Locus	Traits governed	Reference
Wheat (*Triticum aestivum* L.)	*Kna1*	Controls the selectivity of Na^+^ and K^+^ transport from root to shoot and maintains high K^+^/Na^+^ ratio	Gorham et al. (1990), Dubcovsky et al. (1996)
	*Nax1*	Both are involved in decreasing Na^+^ uptake and enhancing K^+^ loading into the xylem	Lindsay et al. (2004), Huang et al. (2006)
Rice (*Oryza sativa* L.)	*qRL-7, qDWRO-9a* and *qDWRO-9b qBI-1a* and *qBI-1b*	Play important roles in root length and root dry weight at seedling stage under saline conditions	Sabouri and Sabouri (2008)
	*QNa, QNa:K, SKC1/OsHKT8*	Regulate K^+^/Na^+^ homoeostasis	Ren et al. (2005)
	*qDM-3* and *qDM-8, qSTR-6*	Improve Na^+^/K^+^ ratio under saline conditions	Sabouri (2009)
	*qNAK-2* and *qNAK-6*	Improve Na^+^/K^+^ ratio	Yao et al. (2005)
	*Saltol*	Controls shoot Na^+^/K^+^ homoeostasis	Thomson et al. (2010)
	*Saltol* and *non-Saltol*	Control shoot Na^+^/K^+^ homoeostasis	Alam et al. (2011)
	*QKr1.2*	Controls K^+^ content in root	Ahmadi and Fotokian (2011)
Barley (*Hordeum vulgare*)	Five QTL for ST were identified on chromosomes 1H, 2H, 5H, 6H, and 7H, which accounted for more than 50% of the phenotypic variation	Enhance vegetative growth under saline stress	Zhou et al. (2012)
	A locus *HvNax3* on the short arm of chromosome 7H in wild barley (*Hordeum vulgare* ssp. *spontaneum*) accession CPI-71284-48	Reduces shoot Na^+^ content by 10–25% in plants grown under salt stress (150 mM NaCl)	Shavrukov et al. (2010)
White clover (*Trifolium repens* L.)	Several QTLs for ST, some at common locations, but each of low scale	Affect ST during vegetative stage	Wang et al. (2010)
Soybean (*Glycine max* (L.) Merr.)	A major QTL for ST was identified near the Sat091 SSR marker on linkage group (LG) N	Maintains growth under salt stress	Lee et al. (2004)
	Eight QTLs for ST were detected	Maintains growth under salt stress	Chen et al. (2008)
	A major QTL for ST was detected	Maintains healthy growth under salt stress	Hamwieh et al. (2011)

In hexaploid bread wheat (*Triticum aestivum*), important locus (*Kna1*) has been reported that regulate the transport of Na^+^/K^+^ from root to shoot specifically, by containing a lower Na^+^/K^+^ ratio within the leaves (Gorham et al., 1987, 1990; Dubcovsky et al., 1996; Luo et al., 1996). Meanwhile, in durum *Triticum turgidum* L. ssp. durum Desf. (wheat) discharge process of Na^+^ is linked to *Nax1* (Na^+^ exclusion 1; Huang et al., 2006, 2008), that might be related to the HKT8 (HKT1;5) and HKT7 (Na^+^ transporters HKT1;4). It has been reported that *Nax1* loci efficiently reduce Na^+^ passage to shoot from root, by keeping Na^+^/K^+^ balance within the leaf of wheat by loading K^+^ into and excluding Na^+^ from, the xylem of the plant (James et al., 2006). Using F2 population of a hybrid within indica rice cultivar ‘IR36’ and japonica rice cultivar ‘Jiucaiqing,’ two QTLs identified for root Na^+^/K^+^ ratio, which were mapped to chromosomes 2 and 6 (Yao et al., 2005). For Salt tolerance traits different QTLs have been recognized in rice which include those at chromosome number 1*- Saltol* QTL, *QNa*, and *SKC1/OsHKT8* along with, QNa:K on chromosome 4. *Saltol* describes many changes for the uptake of ion during salinity stress (Bonilla et al., 2002; Gregorio et al., 2002). For highest Na^+^ uptake *QNa* is QTL (Flowers et al., 2000). For Na^+^/K^+^, QNa:K is the corresponding QTL (Singh et al., 2001). For regulation of K^+^/Na^+^ ratio for homoeostasis in salt stress tolerant indica cultivar ‘Nona Bokra’ *SKC1/OsHKT8* is the corresponding QTL (Lin et al., 2004; Ren et al., 2005). Also, many other QTLs are on the every chromosome except chromosome nine in the root for Na^+^/K^+^ ratio, and for exchange of ion three QTLs on chromosomes 10 and 3 (Sabouri and Sabouri, 2008), for tissue Na^+^/K^+^ ratio four QTLs and each for Na^+^ and K^+^ uptake on various chromosomes one QTL (Lang et al., 2001). Thereafter, 14 QTLs was identified for shoot and root Na^+^/K^+^ ratio and Na^+^ and K^+^ content on different rice chromosomes, recently (Ahmadi and Fotokian, 2011). Among these QTLs, on chromosome 1 for root K^+^ content, *QKr1.2* was identified as one of very bright QTL as it explained around 30% of the variation of observed salt stress tolerance in rice. Furthermore, on rice chromosomes 8 and 10, two newly identified QTLs (*SalTol*8-1 and *SalTol*10-1) based on an F2 hybrid of a cross between a high salt stress tolerant line (IR61920-3B-22-2-1) and a medium salt stress tolerant line (BRRI-dhan40; Islam et al., 2011).

Also, in *Hordeum vulgare* L. (barley, **Table 1**), many studies have discovered QTLs for salt stress tolerance related phenotypes. Recently, 30 QTLs were identified for 10 different traits, such as K^+^ and shoot Na^+^ content, yield-related traits, several growth and Na^+^/K^+^ ratio, in populations grown on normal soil and salt affected soil. In the three species of *Helianthus* sp. (sunflower) and *Helianthus paradoxus*, ion-uptake traits related QTL analysis from highly salt affected habitat and its relative ancestor *H. petiolaris* and *H. annuus* which are both relatively salt sensitive, identified 14 ion uptake QTLs (Lexer et al., 2003). Additional studies are required to decide the benefits of unreported QTLs within crop breeding to improve salt stress tolerance. Since molecular marker techniques for breeding is economical and rapid, this technique is a very powerful method to enhance breeding programs to improve plant tolerance toward salinity. Especially, DNA markers are very important in plant breeding for the selection of polygenic traits, because of the absence of genotype X environment interaction, epistatic effect, and also ease in the picking up of homozygous plants and the homozygous lines can be greatly distinguished from the others at an early generation. Before the crosses of parental lines, molecular characterization of germplasm can help the genetic variations among the parental genotypes increase. Genetic diversity present in the breeding population is maximized and the labor time that is required for either direct selection in traditional breeding or in direct selection through QTLs minimizes. Even though this kind of procedure remains encouraging, its implementation to the complex traits such as salt stress tolerance may be limited due to the close genetic relationship between, wide confidence intervals and, big sample size requirement for screening of the segregating populations, parental population, and possible interactions between genotype and environment for QTL study.

## Available Marker Systems in *Brassica*

In *Brassica*, genome research with the application of marker assisted program began to emerge in the late 1980s when the first RFLP linkage map for *B. oleraceae* (Slocum et al., 1990), *B. napus* (Landry et al., 1991), and *B. rapa* (Song et al., 1991) was developed. For phylogenetic studies and genetic mapping in *Brassica*, RFLPs and RAPDs have been extensively used (Williams et al., 1990). However, the discovery of the PCR (Mullis and Faloona, 1987) leads the potential to increase the variety and density of marker in the already existing genetic maps with ISSR, AFLP and with the microsatellites (Grist et al., 1993), also called as SSR. SSRs are highly important resource of map-based alignment among distinct crosses, because of their robust, simple, and relatively inexpensive analysis and highly polymorphic nature. The number of available *Brassica* SSRs (microsatellite) primers is increasing (http://www.brassica.info/ssr/SSRinfo.htm) the list is given in the **Table 2**. *Brassica* genome integration greatly assisted the release of highly polymorphic mapped based, robust SSR markers of the entire *B. nigra*, *B. rapa*, *B. napus*, and *B. oleracea* genome into public domain. A large number of SSRs (microsatellite) markers have been developed among the cultivated *Brassica* species such as *B. oleracea* (AACC) and the diploids *B. rapa* (AA), *B. nigra* (BB), and *B. oleracea* (CC) which have been shown to be applicable within and between different *Brassica* species. One of the main limit to develop SSR markers in some *Brassica* crops is the lack of finished genome sequence. However, thanks to development of sequencing technology, *B. rapa, B. oleracea*, and *B. napus* are sequenced, recently (Wang et al., 2011; Yu et al., 2013; Shi et al., 2014; Sharma et al., 2015). From this sequence, 140998, 229389, 420991 mono- to hexanucleotide repeat microsatellites are identified using PERL5 script MIcroSAtellite (Thiel et al., 2003). From these identified microsatellites, 115869, 185662, and 356522 SSR markers were developed using *in silico* method, respectively (Shi et al., 2014; **Table 3**). In the past few years, the research work has clearly proven the power of candidate gene studies and genetic maps of high density for the location of molecular markers that are closely linked with the useful trait (s) within *Brassica* have been developed and most of them have been successfully integrated into the *Brassica* oilseed breeding programs (**Table 4**).

**Table 2:** The number of available *Brassica* microsatellite primers in public domain.

Microsatellite set	No. available in public domain
BBRC microsatellite program	397
HRI set	6
Kresovich and Szewc-McFadden	24
Lagercrantz et al. (1993)	5
AAFC Consortium (commercial)	80
Suwabe et al. (2002, 2003)	38
Uzunova and Ecke (1999)	8
Bell and Ecker (1994; *Arabidopsis* microsatellites)	30
INRA Versailles (*Arabidopsis* microsatellites)	120
Celera Consortium (commercial)	171
Oilcrops (http://oilcrops.info//SSRdb)	3974
**Total**	4853

**Table 3:** Microsatellite sequence from the genome of *Brassica rapa, B. oleracea, B. napus* (Shi et al., 2014), and *Arabidopsis thaliana* (*Arabidopsis* Genome Initiative, 2000).

Motif	*B. napus*		*B. oleracea*		*B. rapa*		*A. thaliana*	
	Minimum repeat number	Number (%)	Minimum repeat number	Number (%)	Minimum repeat number	Number (%)	Minimum repeat number	Number (%)
Mono	12	97 128 (23.1)	12	55 433 (24.2)	12	31 258 (22.2)	12	13 650 (43.4)
A	12	94 281 (22.4)	12	52 021 (22.7)	12	29 536 (20.9)	12	13 434 (42.7)
C	12	2847 (0.7)	12	3412 (1.5)	12	1722 (1.2)	12	216 (0.7)
Di	6	98 816 (23.5)	6	55 336 (24.1)	6	33 885 (24.0)	6	7433 (23.6)
AT	6	57 070 (13.6)	6	33 315 (14.5)	6	19 697 (14.0)	6	852 (2.7)
AG	6	34 638 (8.2)	6	18 593 (8.1)	6	11 683 (8.3)	6	2034 (6.5)
AC	6	7072 (1.7)	6	3411 (1.5)	6	2490 (1.8)	6	4544 (14.4)
CG	6	36 (0.0)	6	17 (0.0)	6	15 (0.0)	6	3 (0)
Tri	4	91 448 (21.7)	4	47 716 (20.8)	4	32 387 (23.0)	4	9479 (30.1)
AAG	4	29 395 (7.0)	4	15 322 (6.7)	4	9796 (6.9)	4	1435 (4.6)
AAT	4	17 722 (4.2)	4	9355 (4.1)	4	6334 (4.5)	4	3304 (10.5)
ATC	4	12 314 (2.9)	4	6093 (2.7)	4	4211 (3.0)	4	717 (2.3)
AAC	4	10 046 (2.4)	4	5036 (2.2)	4	3637 (2.6)	4	611(1.9)
AGG	4	8349 (2.0)	4	5425 (2.4)	4	3243 (2.3)	4	411 (1.3)
ACC	4	5969 (1.4)	4	2788 (1.2)	4	2144 (1.5)	4	120 (0.4)
AGC	4	2878 (0.7)	4	1390 (0.6)	4	1127 (0.8)	4	509(1.6)
ACT	4	1754 (0.4)	4	818 (0.4)	4	674 (0.5)	4	677 (2.2)
CCG	4	1566 (0.4)	4	799 (0.3)	4	626 (0.4)	4	1633 (5.2)
ACG	4	1455 (0.3)	4	690 (0.3)	4	595 (0.4)	4	62 (0.2)
Tetra	3	91 268 (21.7)	3	48 394 (21.1)	3	29 433 (20.9)	4	462 (1.5)
AAAT	3	33 903 (8.1)	3	18 856 (8.2)	3	11 870 (8.4)	4	98 (0.3)
AAAG	3	10 795 (2.6)	3	5697 (2.5)	3	3496 (2.5)	4	94 (0.3)
AAAC	3	9717 (2.3)	3	4723 (2.1)	3	3333 (2.4)	4	73 (0.2)
AATT	3	7863 (1.9)	3	4088 (1.8)	3	2534 (1.8)	4	24 (0.1)
AATC	3	5112 (1.2)	3	2956 (1.3)	3	1137 (0.8)	4	47 (0.1)
Others	3	23 878 (5.7)	3	12 074 (5.3)	3	7063 (5.0)	4	126(0.4)
Penta	3	29 058 (6.9)	3	15 012 (6.5)	3	9856 (7.0)	4	283 (0.9)
AAAAT	3	7617 (1.8)	3	4051 (1.8)	3	2758 (2.0)	4	14 (0)
AACCG	3	3541 (0.8)	3	2169 (0.9)	3	1000 (0.7)	4	8 (0)
AAAAC	3	2424 (0.6)	3	1186 (0.5)	3	878 (0.6)	4	34 (0.1)
AAAAG	3	2351 (0.6)	3	1164 (0.5)	3	683 (0.5)	4	25 (0.1)
AAATT	3	1488 (0.4)	3	784 (0.3)	3	568 (0.4)	4	2 (0)
AAACC	3	1273 (0.3)	3	713 (0.3)	3	490 (0.3)	4	10 (0)
AATAT	3	1083 (0.3)	3	573 (0.2)	3	394 (0.3)	4	1 (0)
Others	3	9281 (2.2)	3	4372 (1.9)	3	3085 (2.2)	4	189 (0.6)
Hexa	3	13 273 (3.2)	3	7498 (3.3)	3	4179 (3.0)	4	149 (0.5)
AAAAAT	3	1803 (0.4)	3	987 (0.4)	3	628 (0.4)	4	2 (0)
AAAATT	3	1341 (0.3)	3	776 (0.3)	3	267 (0.2)	5	1 (0)
AAAAAC	3	746 (0.2)	3	395 (0.2)	3	269 (0.2)	4	7 (0)
AAAAAG	3	644 (0.2)	3	357 (0.2)	3	175 (0.1)	4	4 (0)
AAATAT	3	436 (0.1)	3	246 (0.1)	3	150 (0.1)	-	0 (0)
Others	3	8303 (2.0)	3	4737 (2.1)	3	2690 (1.9)	4	135 (0.4)
**Total**	3	420991 (100)	3	229389 (100)	3	140998 (100)	4	31456 (100)

*Arabidopsis thaliana microsatellite sequence was found from using msatcommander software (Faircloth, 2008). This table only shows particular sequence motifs data with whole motifs up to hexanucleotide is in Supplementary Table S1.*

**Table 4:** Study of important traits in *Brassica* species.

Character	Trait	Species	Reference
Morphological traits	Seed coat color	*B. napus*	Somers et al. (2001)
“	“	*B. juncea*	Padmaja et al. (2005)
“	“	*B. rapa*	Mukhlesur et al. (2007)
	Seed coat color	*B. napus*	Rezaeizad et al. (2011)
	Seed coat color	*B. napus*	Qu et al. (2015)
	Seed coat color	*B. napus*	Gustafson et al. (2006), Fu et al. (2007), Xiao et al. (2007), Francki et al. (2010)
“	Yield influencing QTLs	*B. juncea*	Ramchiary et al. (2007)
“	Flowering time	*B. napus*	Ferreira et al. (1995b)
“	Root morphology	*B. rapa*	Lu et al. (2008)
“	Plant height	*B. napus*	Foisset et al. (1995), Barret et al. (1998)
“	Petal – less flower	*B. napus*	Fray et al. (1997)
			
Oil content	Glucosinolate content	*B. napus*	Uzunova et al. (1995)
“	Oleic acid	*B. napus*	Hu et al. (1999)
“	Linoleic acid	*B. napus*	Somers et al. (1998), Hu et al. (2006)
“	Linolenic acid	*B. napus*	Hu et al. (1995), Tanhuanpää and Schulman (2002)
“	Erucic acid	*B. napus*	Ecke et al. (1995)
“	“	*B. juncea*	Gupta et al. (2004)
“	Seed glucosinulates	*B. napus*	Uzunova et al. (1995), Howell et al. (2003)
	Oil content	*B. napus*	Qu et al. (2015)
	Fiber content	*B. napus*	Gustafson et al. (2006), Fu et al. (2007), Xiao et al. (2007), Francki et al. (2010)
	Glucosinolate content	*B. napus*	Hasan et al. (2008)
			
Disease resistance	*Albugo candida*	*B. napus*	Ferreira et al. (1995c), Kole et al. (2002b)
“	*Plasmodiophora brassicae*	*B. pekinensis*	Lee et al. (2002)
“	*Leptosphaeria maculans*	*B. napus*	Dion et al. (1995), Ferreira et al. (1995a), Pilet et al. (1998a,b, 2001)
“	Turnip mosaic virus	*B. napus*	Walsh et al. (1999), Dreyer et al. (2001)
“	*Sclerotiana sclerotiorum*	*B. napus*	Zhao and Meng (2003)
“	*Phoma*	*B. napus*	Plieske and Struss (2001b)
“	*Pyrenopeziza brassicae*	*B. napus*	Pilet et al. (1998b)
			
Abiotic stress	Winter survival	*B. napus*	Kole et al. (2002a), Asghari et al. (2007)
	Drought and cold	*B. napus*	Kagale et al. (2007)
			
Male sterility	‘Ogura’ fertility restorer	*B. napus*	Delourme et al. (1998), Brown et al. (2003)
“	‘Polima’ fertility restorer	*B. napus*	Jean et al. (1998)
“	‘Kosena’ fertility restorer	*B. napus*	Imai et al. (2003)
“	*tournefortii* fertility restorer	*B. napus*	Janeja et al. (2003)

## Importance of Microsatellite (SSR) Marker

Over the past few years, various new PCR based marker such as AFLPs, RAPDs, and microsatellites have been developed and applied in crop improvement program. Microsatellites markers have great deal of potential among all the markers. Litt and Luty (1989) coined the term microsatellite. Microsatellites markers are also defined as simple sequence repeats (SSRs) which are based on unique DNA sequences that are flanking short repetitive traits of simple sequence motifs, for example – di or tri nucleotides. They are randomly distributed within the eukaryotic genomes (Smith and Devey, 1994). They are variable with respect to the number of repeats, pedigree analysis and are highly efficient in the fingerprinting and show co-dominant inheritance of different crops. The extra ordinary level of instructive polymorphism at SSR locus originated from the apparent tendency of development or replication or unequal crossing-over event at the time of meiosis. The strengths of SSR markers include their high numbers in eukaryotes, the codominance of alleles, and their arbitrary dispensation throughout the genome with special consortium with in low-copy regions (Morgante et al., 2002). Also, low quantities of template DNA (10–50 ng/reaction) are required, because of the PCR based technique. Reproducibility of SSR markers is high due to the use of lengthy PCR primers (Im et al., 2014b), and its use does not even require high quality DNA. Even though, the RFLP was one of the first markers used for genome analysis, RFLP technique is laborious and RFLP is less polymorphic than SSR marker. Also, improved technique that is more simple and efficient to find polymorphism in SSR marker makes SSR marker more useful (Kumar et al., 2015). Furthermore, since conventional microsatellite generating method using genomic libraries (Weising et al., 2005; Smykal et al., 2006) was replaced by *in silico* microsatellite generating method, many software generating microsatellite was made, such as MISA, MicrosatDesign, msatminer, msatcommander, IMEx, WebSat (Thiel et al., 2003; Singan and Colbourne, 2005; Thurston and Field, 2005; Mudunuri and Nagarajaram, 2007; Faircloth, 2008; Martins et al., 2009). These programs and genome sequence information make SSR marker generation procedure convenient. Therefore, the number of available SSR markers increases rapidly and available SSR marker in the genome becomes more dense. Therefore, the amount of SSR marker database is increasing. Even though, there are not so many reports about QTL for salt stress tolerance, many QTL has been identified and QTL information was enhanced by association mapping using SSR markers in *Brassica* crops. In *Brassica napus*, 53 SSR markers were found to be significantly associated to three phenolic fractions and 11 markers found to be associated to total phenolic acid contents. Among these markers, four SSR markers are derived from QTL for seed color (Rezaeizad et al., 2011). Twenty five and 11 SSR markers were found to be associated with seed coat color and oil content. Among these markers six SSR markers are associated with both of coat color and oil content (Qu et al., 2015). Also, association study between known major QTL and SSR marker is useful to find candidate gene because of high density of SSR marker in genome. Main QTL for seed color and fiber content on one of the homoeolog chromosomes A9 or C8 in *B. napus* has been described by many studies (Gustafson et al., 2006; Fu et al., 2007; Xiao et al., 2007). SSR markers bridge the sequence contig overlaying this QTL was identified and four of these SSR markers from small genomic region less than 50 kb which are strongly associated with seed color and fiber content traits were identified (Francki et al., 2010). These SSR markers in known major QTL or near these QTL which are strongly associated with the trait are useful to find candidate gene. SSR markers can be analyzed interspecifically. After mapping SSR markers in several species, comparative mapping of SSR markers can be made using alignment of orthologous loci (Yu et al., 2004; Zhang et al., 2007; Yu and Li, 2008). This will help in identifying the origin of QTL, find candidate gene as well as structural collinearity in genome between two species can also be dissected out.

## The Candidate Gene Approach

Many traits of agricultural importance, which includes salinity tolerant traits, exhibits quantitative inheritance, which is mostly the result of multiple genes influenced by the environment. Due to the their imprecise localization on the genetic maps and multiplicity of genes defining a quantitative trait and their incomplete effects on phenotypic differentiation, the candidate gene approach is more adapted to the QTL characterization. The candidate gene approach has been emphasized as an encouraging method for combining QTL analysis with the large-scale data available on the cloning and genes characterization (Pflieger et al., 2001). Genes likely to be involved in the biochemical pathways, in this technique, that lead to a trait articulation, are engaged as molecular markers for QTL analysis. The *Brassica* species are the closest crop relative of the model crucifer *Arabidopsis thaliana* and the complete sequencing of this model crop has also created the way to relative examination into the complicated structure of *Brassica* genomes (Chalhoub et al., 2003). The comparative studies of flanked genome regions of known genes shows the extensive co-linearity between *Arabidopsis* and *Brassica* genome segments on a small scale level (Fourmann et al., 1998; Babula et al., 2003). Over the long chromosome stretches, the large scale synteny makes way to use the sequence data from the markers bound QTLs or genes of interest in *Brassica* to determine candidate genes from the chromosome segment in *Arabidopsis*. For example, different homeologs regions in *B. napus* and *B. rapa* that have different QTLs that regulate flowering time, each show useful similarity to the *Arabidopsis* chromosomal part containing a particular number of genes that influence the flowering time (Ferreira et al., 1995b; Lagercrantz et al., 1996).

Furthermore, comparative mapping between *Brassica* and *Arabidopsis* with SSR marker is helpful to identify candidate gene. Comparative mapping based on SSR marker between *B. rapa* and *A. thaliana* shows corresponding regions in *A. thaliana* for *Crr1* and *Crr2* which are QTL for club root resistance in *B. rapa* are in a small region of *A. thaliana* chromosome four where one of the region of disease resistance gene cluster has been identified. Therefore, it seems that the gene for club root resistance in *B. rapa* is related with disease resistance gene cluster in *A. thaliana* (Suwabe et al., 2006).

While comparing the salt stress tolerance of a particular *Brassica* species at the early growth stages, *B. carinata*, *B. juncea*, and *B. napus*, had better salinity tolerance than *B. campestris* (Ashraf and McNeilly, 1990). The reactions of four *Brassica* species, *B. campestris*, *B. carinata*, *B. juncea*, and *B. napus* to four different salts, CaCl_2_, MgCl_2_, NaCl, and Na_2_SO_4_, was tested at the seedling and germination stage using sand culture and solution (Ashraf et al., 1989). Effect of NaCl was more significantly within the effect of four salts and it inhibits the germination rate of all four species. There was no uniform connection between results for seedling growth and germination rate, exceptions in *B. napus*, which showed more seedling growth and better germination rate under the salinity stress as compared to the other three species.

Huge and still growing *Arabidopsis* EST database and the amalgamation into the comparative *Brassica* genome study helps fine mapping of the genomic re-arrangements and the recognition of regions that contains genes crop plants. Also recognition of the association of given traits in *Brassica* crops with the candidate genes of *Arabidopsis* and the generation of the molecular markers that have association with the corresponding genes (Panjabi et al., 2008). For example the Co (constans) gene isolated from *Arabidopsis*, which is involved in late flowering is a putative candidate gene (genes isolated in model species establish the putative CGs for the agronomic species) for two QTLs, which control the flowering time in *B. napus* (Putterill et al., 1995; Robert et al., 1998). Putative CGs involved in fatty acid metabolism, were mapped on the rapeseed genome. In *B. rapa*, a important QTL for flowering time was found in the region homologous to the *Arabidopsis* chromosome five top, where many genes that regulate flowering are located (Kole et al., 2001). In *B. napus*, many cold and drought induced genes were isolated and characterized and there is a huge correlation between the development of freezing tolerance and the expression of some of these genes, which seems to be up-regulated by cold stress (Kole et al., 2002a; Asghari et al., 2007; Kagale et al., 2007).

In *B. oleracea*, fifteen QTLs regulating the flowering time were situated to the *Arabidopsis* genomic segments that contain flowering time genes that affect flowering (Okazaki et al., 2007). Similarly large sets of *Arabidopsis* QTLs were found for *Brassica* QTLs that were influencing leaves and whole plant structure (Lan and Paterson, 2001). Also, based on synteny, SSR markers near the QTL for glucosinolate content in *B. napus* whose orthologs in *A.thaliana* is linked to candidate genes were identified and four putative candidate genes for glucosinolate biosynthesis were identified (Hasan et al., 2008.). Furthermore, Using *in silico* analysis about *B. oleracea* genome and synteny with *A. thaliana*, putative seven candidate major loci for regulating glucosinolate content were proposed (Sotelo et al., 2014.). Due to this *Arabidopsis* genome sequence is a very informative resource for identifying and further assessment of candidate genes that may account for the control of complicated traits in *Brassica* at the genetic level. But main difficulty in the application of genetic information of *Arabidopsis* for the map-based cloning, candidate gene identification and marker development in *Brassica* crop species is hindered by the complicated structure of the polyploidy *Brassica* genome.

## Conclusion

Today’s agriculture certainly requires salt tolerant *Brassicas* for the very commercial purpose of the crop. Recent in-depth investigations at physiological and moleuclar levels have identified many ways by which wild type plants cope with salinity stress. Thanks to close relationship and the significant inter and intra specific variation within *Brassica* species which shows huge potential for breeding for salt stress tolerance in *Brassica* crops. Nonetheless, it is clear that to link the salt tolerance trait with QTL position on the chromosome, a proper breeding program assisted with the markers is a prerequisite.

Among the various molecular markers available for this purpose, SSRs are gaining huge attention. SSR markers have many advantages such as high polymorphism, relatively simple methods for identification and most importantly, small amounts of plant material is required for the PCR-based experiment. Other advantages of SSR markars are their random distribution, moderate genome coverage and co-dominant inheritance.

Since *Brassica* and *Arabidopsis* belongs to the same family, many studies and database of *Arabidopsis* are helpful to do breeding in order to improve salt tolerance. Although genome of B. Rapa (485 Mb) and B. Oleracea (630 Mb) is bigger than the genome of A. *thaliana* (Wang et al., 2011; Yu et al., 2013) and *A. thaliana* genome segments are dispersed and rearranged in many *Brassica* crops (Kowalski et al., 1994; Lagercrantz, 1998; Parkin et al., 2005), it has many genomic segments which are highly conserved. Therefore, comparative molecular marker mapping is quite informative. There are 140998, 229389, 420991 mono to hexanucleotide repeat SSRs from the genome of *B. rapa, B. oleracea*, and *B. napus*, respectively, out of which 31456 are microsatellite sequence which are candidates of SSR markers from the genome of *A. thaliana* (**Table 3**, Supplementary Data S1). There is tendancy that major motif sequences of candidates of SSR marker in both *Brassica* crops and *A. thaliana* are A/T rich and the motif sequences that are abundant in the genome of *Brassica* crops are also abundant in *A. thaliana*. Even microsatellite sequence in *A. thaliana* genome is much less than that in *Brassica* crops, the number of SSR marker is enough to show potential of comparative study of salt tolerance QTL using SSR marker in *Brassica* crops and *A. thaliana*. Furthermore, since EST-SSR marker developed from EST-database is more useful to find the candidate genes, the available huge EST-database is helpful for identifying the candidate gene from the QTL. Also, other physiological and agronomical traits can be studied with SSR markers to make a robust and healthy plants with high yield.

## Conflict of Interest Statement

The authors declare that the research was conducted in the absence of any commercial or financial relationships that could be construed as a potential conflict of interest.
